# A Novel Drug Resistance Mechanism: Genetic Loss of Xeroderma Pigmentosum Complementation Group C (*XPC*) Enhances Glycolysis-Mediated Drug Resistance in DLD-1 Colon Cancer Cells

**DOI:** 10.3389/fphar.2019.00912

**Published:** 2019-09-10

**Authors:** Yu Han, Yuan Qing Qu, Simon Wing Fai Mok, Juan Chen, Cheng Lai Xia, Hu Qiang He, Zheng Li, Wei Zhang, Cong Ling Qiu, Liang Liu, Betty Yuen Kwan Law, Vincent Kam Wai Wong

**Affiliations:** ^1^State Key Laboratory of Quality Research in Chinese Medicine, Macau University of Science and Technology, Macau, China; ^2^The Key Laboratory of Molecular Biology of Infectious Diseases designated by the Chinese Ministry of Education, Chongqing Medical University, Chongqing, China; ^3^Foshan Maternal and Child Health Research Institute, Foshan Women and Children’s Hospital Affiliated to Southern Medical University, Foshan, China

**Keywords:** *BAX*, *BAK*, *XPC*, glycolysis, drug-resistance, CRC

## Abstract

The pro-apoptotic proteins BAX and BAK are critical regulatory factors constituting the apoptosis machinery. Downregulated expression of BAX and BAK in human colorectal cancer lead to chemotherapeutic failure and poor survival rate in patients. In this study, isogenic DLD-1 colon cancer cells and the *BAX* and *BAK* double knockout counterpart were used as the cellular model to investigate the role of BAX/BAK-associated signaling network and the corresponding downstream effects in the development of drug resistance. Our data suggested that DLD-1 colon cancer cells with *BAX*/*BAK* double-knockout were selectively resistant to a panel of FDA-approved drugs (27 out of 66), including etoposide. PCR array analysis for the transcriptional profiling of genes related to human cancer drug resistance validated the altered level of 12 genes (3 upregulated and 9 downregulated) in DLD-1 colon cancer cells lack of *BAX* and *BAK* expression. Amongst these genes, *XPC* responsible for DNA repairment and cellular respiration demonstrated the highest tolerance towards etoposide treatment accompanying upregulated glycolysis as revealed by metabolic stress assay in DLD-1 colon cancer cells deficient with *XPC*. Collectively, our findings provide insight into the search of novel therapeutic strategies and pharmacological targets to against cancer drug resistance genetically associated with *BAX*, *BAK*, and *XPC*, for improving the therapy of colorectal cancer *via* the glycolytic pathway.

## Introduction

Colorectal cancer (CRC) is one of the most common malignant tumor types (Liu and Liu, 2018), accounting for approximately 10% of global cancer deaths (Gandomani et al., 2017). Although conventional chemotherapy has demonstrated satisfactory therapeutic efficacy in the treatment of CRC, a considerable amount of patients are still suffering from spontaneous or acquired resistance after chemotherapy, which significantly reduced the survival rate (Hammond et al., 2016). Therefore, it is essential to clarify the transcriptional and molecular machinery that drives the transition of CRC from drug-sensitive phenotype to the resistant counterpart for facilitating the search of novel pharmacological targets and drug candidates.

Mechanisms underpinning the chemotherapy resistance in cancer cells are varied (Holohan et al., 2013). For instance, overexpression of the DNA repair mediator, xeroderma pigmentosum complementation group C (*XPC*) (Sugasawa et al., 1998; Shell et al., 2013), enhances the repairment of damaged DNA *via* nucleotide excision repair (NER) process in cancer cells, rendering them the capability of escaping from cytotoxicity induced by chemotherapeutic drugs (Fautrel et al., 2005). In terms of regulation of cellular respiration, the reduced protein level of XPC in human cancer cells may result in a metabolic shift from mitochondrial oxidative phosphorylation (OXPHOS) to anaerobic glycolysis in response to the accumulation of nuclear DNA damage and increased oxidant production (Mori et al., 2017). Such alteration of energy metabolism implicated that production of energy rapidly to maintain the continuity of the biological process, therefore, supporting the high proliferative rate of cancer cells even in the drastic environment with insufficient oxygen supply (Heiden et al., 2009; Najafov and Alessi, 2010). The upregulation of glycolytic pathway is generally observed in most cancer cells which are associated with their worsened responses towards common anticancer agents as reflected from the restored sensitivity upon the inhibition of glycolysis (Xu et al., 2005; Pelicano et al., 2006). For example, the overexpression of glucose transporters in human colon cancer cells lead to high-rate glycolysis under hypoxia, which inhibits apoptosis and promotes cellular survival, thereof, the development of drug resistance (Cao et al., 2007). On the other hand, deficient in apoptosis is another molecular culprit of drug resistance as a result of the uncontrolled proliferation of cancer cells (Helmbach et al., 2002). As such, the promotion of apoptosis is currently one of the main therapeutic strategies for the pharmacological intervention of cancers (Wong, 2011). The pro-apoptotic proteins BAX and BAK are important to the induction of apoptosis in cancer cells *via* the interaction with mitochondrial voltage-dependent anion channels and induction of downstream cytochrome c release (Shimizu et al., 1999; Gerl and Vaux, 2005). However, persistent chemotherapy may stimulate mutation and loss of functions of these genes which hampers the therapeutic effects (Housman et al., 2014). In fact, BAX and BAK expression are severely attenuated in many malignancies, and downregulation of *BAX* and *BAK* are associated with the development of apoptosis resistance (Wei et al., 2001). We previously found that double knockout of *Bax* and *Bak* in mouse embryo fibroblasts (MEFs) were resistant to apoptosis induced by a panel of chemotherapeutic agents, such as cisplatin, doxorubicin, paclitaxel, etoposide, and staurosporine (Law et al., 2014; Law et al., 2016). Of note, the protein function of BAX and BAK can also be inactivated in cancer cells under the cellular environment dominated by anaerobic energy metabolism conferring survival advantages to cancer growth (Tomiyama et al., 2006).

In this study, the effect of deficiency of both *BAX* and *BAK* on the transcription profiling in DLD-1 colon cancer cells was examined. Owing to the significance of *BAX* and *BAK* in the induction of apoptosis and the complexity of the involved downstream signaling network, we aimed to identify the genes potentially regulated by *BAX* and *BAK*. The alteration of such genes may manipulate the apoptotic process of DLD-1 colon cancer cells and implicate in the development of drug resistance. This hypothesis was tested by screening the gene expression profile in wild type DLD-1 colon cancer cells (DLD-1 WT cells) and the isogenic line ablated with both *BAX* and *BAK* (DLD-1 BAX–BAK DKO cells) using the human cancer drug resistance PCR array. We show here that the expression of *XPC* was downregulated after the loss of *BAX* and *BAK* accompanying a reduction of sensitivity towards etoposide treatment. The in-depth metabolic analysis further verified the enhanced glycolysis in our cellular model after the suppression of *XPC* gene. Therefore, we reported here a new molecular pathway of *BAX*/*BAK*-induced drug resistance of DLD-1 colon cancer cells which is mediated by the downregulated expression of the *XPC* gene *via* the further heightening of glycolytic process.

## Materials and Methods

### Cells Culture

DLD-1 BAX–BAK DKO cells and DLD-1 WT cells were purchased from Sigma-Aldrich (St. Louis, MO, USA). These cell lines were authenticated by ATCC. RPMI 1640 medium (Gibco, Waltham, MA, USA) supplemented with 1% penicillin–streptomycin–glutamine and 10% fetal bovine serum (Gibco, Waltham, MA, USA) was used as the culture medium. For DLD-1 BAX–BAK DKO cells, the culture medium was additionally supplemented with 1% sodium pyruvate (Gibco, Waltham, MA, USA). Cells were cultured at 37°C in a humidified incubator with 5% CO_2_.

### Chemicals, Antibodies, and Small Interfering RNAs

The following chemicals were used at doses indicated in the text and figures. Etoposide and the FDA-approved Drug Library were purchased from Meryer (Shanghai, China) and Selleck (Houston, TX, USA), respectively. Antibodies against CYP2C19, XPC, and PPARα were obtained from Abgent (San Diego, CA, USA). Antibodies against BAX, BAK, NFBKIβ, EGFR, and FOS were purchased from Cell Signaling Technology (Danvers, MA, USA). NAT2 antibody was purchased from OmnimAbs (Alhambra, CA, USA). TPMT antibody was obtained from 4A Biotech (Beijing, China). UGCG antibody was purchased from Proteintech (Chicago, IL, USA). β-actin antibody was purchased from Sigma-Aldrich (St. Louis, MO, USA). siRNAs targeting *EGFR*, *FOS*, *NFKB1*, *PPARα*, *RXRα*, *RXRβ*, *TPMT*, *UGCG*, and *XPC* were obtained from Qiagen (Hilden, Germany). All reagents were purchased from Sigma-Aldrich (St. Louis, MO, USA) unless otherwise specified.

### Real-Time Quantitative PCR

Gene expression was analyzed by real-time quantitative PCR (RT-qPCR) with ViiA™ 7 Real-Time PCR System (Applied Biosystems, Waltham, MA, USA) using PowerUp™ SYBR™ Green Mastermix (ThermoFisher Scientific, San Jose, CA, USA). The cDNA was prepared by using the Transcriptor Universal cDNA Master Kit (Roche, Basel, Switzerland). Primers sequence (see **Table 2**) synthesized by Tech Dragon Ltd. (Hong Kong, China), were designed by employing the ThermoFisher Scientific’s online OligoPerfect™ Designer software and further verified with NCBI’s Primer-BLAST software. Results were normalized to *ACTB* relative to control and analyzed using the ΔΔCt methods (2^-ΔΔCt^).

### Western Blot Analysis

Cells were lysed with RIPA lysis buffer (Cells Signaling Technology, Danvers, MA, USA) for cellular proteins extraction with the concentrations determined by using the Bio-Rad protein assay (Hercules, CA, USA). Proteins from SDS/PAGE was electrotransferred to a Hybond enhanced chemiluminescence nitrocellulose membrane (GE Healthcare, Buckinghamshire, UK) by electrophoresis. The membrane was then immunoblotted with specific antibodies. Proteins detection was performed using Amersham Imager 600 (GE Healthcare, Buckinghamshire, UK) and Clarity Western ECL Substrate (Bio-Rad, Hercules, CA, USA). Band intensities were quantified by using the software Image J (NIH, USA).

### MTT Assay

Cells viability was determined by the half maximal inhibitory concentration (IC_50_) using MTT (3-[4,5-dimethylthiazol-2-yl]-2,5 diphenyl tetrazolium bromide) assay as previously described (Wong et al., 2009). Briefly, cells were seeded and incubated in 96-well plates for treatment with compounds in the FDA-drugs library dissolved in DMSO to different concentrations (0–100 μmol) for 72h. The samples were then incubated with MTT at 37°C for 4 h before the administration of solubilization buffer (10% SDS in 0.01 mol/L HCl) for overnight incubation. Absorbance at *A*_570nm_ was measured to cells viability on a plate reader (Tecan Infinite M200 PRO, Tecan, Männedorf, Switzerland). The percentage of cells viability was calculated using the following formula: Cells viability (%) = (*A*_treated_ – *A*_background_)/(*A*_control_ – *A*_background_) × 100. The drugs resistance fold (RF) was calculated as IC_50 DLD-1 BAX–BAK DKO_/IC_50 DLD-1 WT_.

### RT^2^ Profiler PCR Arrays Analysis

Total RNA was prepared by using the Qiagen RNeasy^®^ Mini Kit (Qiagen, Hilden, Germany) and cDNA was synthesized by the RT^2^ First Strand Kit (Qiagen, Hilden, Germany). Cancer Drug Resistance RT^2^ Profiler PCR Array Kit (Qiagen, Hilden, Germany) comprising 84 cancer drugs resistant or metabolism-related genes were used to evaluate the expression profiling in DLD-1 WT and DLD-1 BAX–BAK DKO cells. The PCR reactions were performed by using RT^2^ SYBR^®^ Green qPCR Mastermix (Qiagen, Hilden, Germany) and the ViiA™ 7 Real-Time PCR System (Applied Biosystems, Waltham, MA, USA). The array data were normalized with a panel of housekeeping gene: *B2M*, *HPRT1*, *RPL13A*, *GAPDH*, and *ACTB*. The integrated web-based software package (Qiagen, Hilden, Germany) was used for data analysis.

### Seahorse XF Metabolic Stress Assay

Cells were plated in XFp Cells Culture Miniplates (Agilent, Santa Clara, CA, USA) at 15000 cells/well. After 24 h, mitochondrial respiration or glycolysis was determined with XFp Cells Mito Stress Test Kit or XFp Cells Glycolysis Stress Test Kit (Agilent, Santa Clara, CA, USA) on Seahorse Bioscience XFp extracellular flux analyzer (Agilent, Santa Clara, CA, USA). The mitochondrial oxygen consumption rate (OCR) was measured by serial injection of oligomycin (10 μmol), carbonyl cyanide 4-(trifluoromethoxy) phenylhydrazone (FCCP) (0.5 μmol), and 0.5 μmol mix of rotenone (complex I inhibitor) and antimycin A (complex III inhibitor). Glucose (10 mmol), oligomycin (10 μmol) and 2-deoxyglucose (2-DG, 50 mmol) were serially injected to measure the extracellular acidification rate (ECAR). Data analysis were performed with Seahorse XFp Analyzer Software (Agilent, Santa Clara, CA, USA).

### LC-MS/MS Measurement of ATP Metabolites

Intracellular ATP, ADP, and AMP contents in the DLD-1 WT and DLD-1 BAX–BAK DKO cells were quantified by LC-MS/MS analysis carried out on a ThermoFisher TSQ LC-MS/MS system (San Jose, CA, USA) using multiple reactions monitoring as reported earlier (Li et al., 2018). Chromatographic separation was performed on the Sepax GP-C18 column at 35°C with the flow rate of 0.2 mL/min. The mobile phase was composed of water (A) and acetonitrile (B). The column was eluted with a linear gradient system: 0–5–10–12–13–18 min, 12–30–40–55–12–12% B. The autosampler was set at 4°C. Mass detection was carried out using ESI in the negative mode using the following optimized parameters: ion spray voltage, 2800 V; vaporizer temperature, 300°C; sheath gas pressure, 50 psi; capillary temperature, 320°C; auxiliary gas pressure, 15 psi. Data acquisition was performed with the Xcalibur software version 2.0.7 (ThermoFisher Scientific, San Jose, CA, USA), and data processing using the Thermo LCquan 2.5.6 data analysis program (ThermoFisher Scientific, San Jose, CA, USA).

## Results

### Genetic Deficiency of the Pro-Apoptotic Proteins BAX and BAK Induced Drug Resistance in DLD-1 Colon Cancer Cells

**Figure 1:**
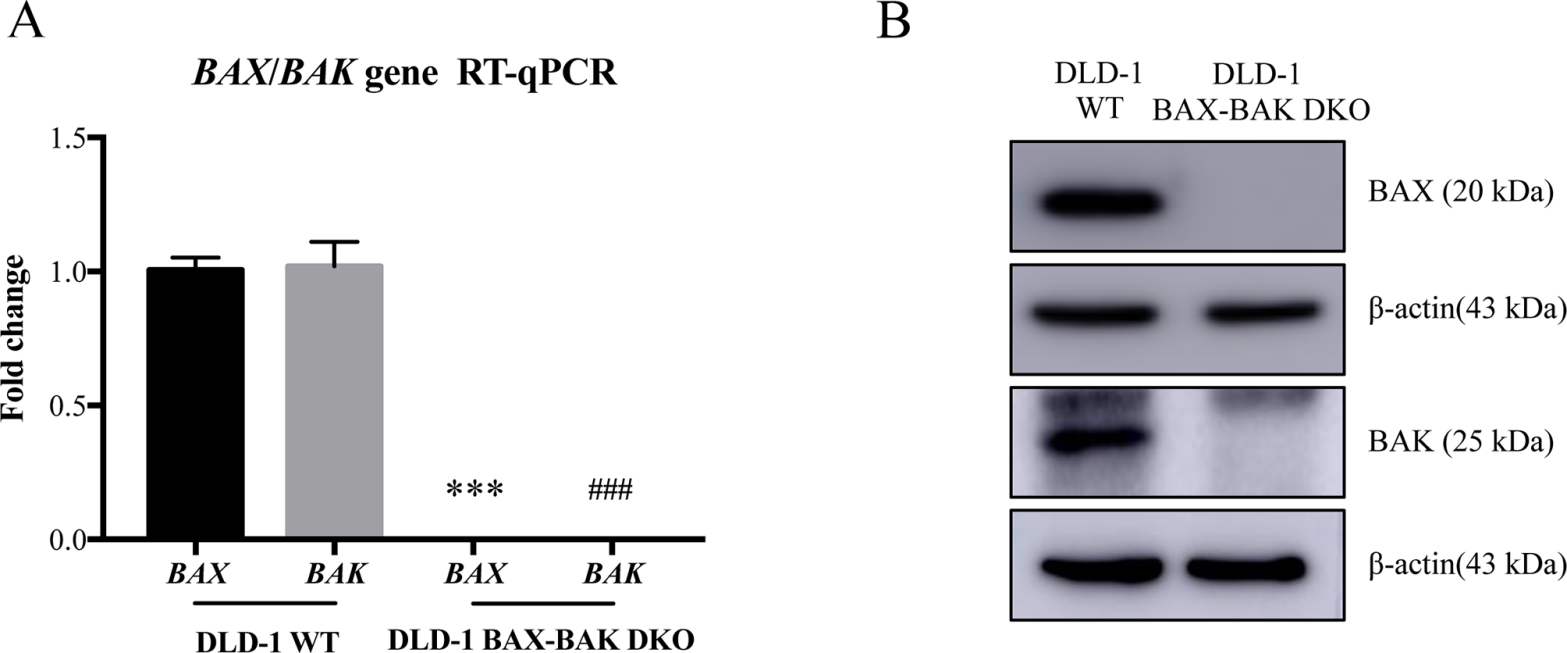
Study of BAX and BAK expression level in DLD-1 colon cancer cells. **(A)** Quantification of *BAX* and *BAK* genes expression in DLD-1 WT and DLD-1 BAX–BAK DKO cells by RT-qPCR. **(B)** Proteins expression levels of BAX and BAK in DLD-1 WT and DLD-1 BAX–BAK DKO cells were detected by Western blot with β-actin as loading control. The full-length images of Western blot are shown in **Figure S1**. The data is represented as the mean ± S.D. (n = 3). ***p < 0.001 and ^###^*p* < 0.001 versus *BAX* and *BAK* expression level in DLD-1 WT cells respectively, *t*-test analysis.

BAX and BAK proteins are critical to the activation of apoptotic pathways mediated by mitochondrial outer membrane permeabilization (O’neill et al., 2016) and the deficiency of *BAX* and *BAK* is related to drug resistance in cancer treatment (Müller et al., 2018). Therefore, cancer cell lines deficient in both *BAX* and *BAK* may serve as ideal models for examining the drug resistance mechanisms. For clarifying the association of BAX and BAK with the development of drug-resistant phenotype of colon cancers, isogenic DLD-1 WT cancer cells and DLD-1 BAX–BAK DKO cancer cells were used as the cellular model in this study. As shown in **Figure 1A**, the expression of both *BAX* and *BAK* in DLD-1 BAX–BAK DKO cells were completely abolished when compared with the DLD-1 WT cells (*p* < 0.001). Consistent with the gene expression study, the BAX and BAK proteins were not detected in DLD-1 BAX–BAK DKO cells as demonstrated by Western blot analysis (**Figure 1B**). We then validated the potency of *BAX* and *BAK* deficiency in the induction of drug resistance in DLD-1 WT and DLD-1 BAX–BAK DKO cells against FDA-approved drugs conventionally used for the therapies of cancer, inflammation and metabolic diseases, as tumorigenesis is closely related to inflammatory disorder and metabolic dysfunction (Coussens and Werb, 2002; Coller, 2014). In total, 234 compounds were chosen from the FDA-approved Drug Library for screening, in which, 66 out of the 234 drugs demonstrated significant cytotoxicity towards the DLD-1 WT and DLD-1 BAX–BAK DKO cells. Notably, DLD-1 BAX–BAK DKO cells were insensitive to 27 out of the 66 drugs (RF ≥ 3) when compared with DLD-1 WT cells (**Table 1**). Therefore, DLD-1 BAX–BAK DKO cells is a reliable drug resistance model with pathways related to the BAX/BAK axis with selectively resistant towards a panel of FDA-approved drugs.

**Table 1:** Drug-resistant potency of FDA-approved anti-cancer drugs in DLD-1 WT and DLD-1 BAX–BAK DKO cells.

Drug name	DLD-1 WT IC_50_ value (µmol)	DLD-1 BAX–BAK DKO IC_50_ value (µmol)	Resistance Fold	Drug name	DLD-1 WT IC_50_ value (µmol)	DLD-1 BAX–BAK DKO IC_50_ value (µmol)	Resistance Fold
Bosutinib	4.78 ± 0.68	18.53 ± 0.67	3.87	Imatinib Mesylate	38.87 ± 0.240	>50	>1.29
Dasatinib	2.15 ± 0.45	>50	>23.25	Pazopanib HCl	30.52 ± 1.732	>50	>1.63
Erlotinib HCl	16.60 ± 5.06	>50	>3	Sorafenib	5.34 ± 0.14	5.79 ± 0.48	1.08
Nilotinib	10.50 ± 0.39	40.65 ± 5.43	3.87	Sunitinib Malate	2.99 ± 0.29	6.24 ± 1.01	2.09
Crizotinib	1.94 ± 0.14	6.80 ± 0.04	3.49	Temsirolimus	32.17 ± 0.17	42.18 ± 1.79	1.31
Docetaxel	0.20 ± 0.09	>10	>50	Vandetanib	5.12 ± 0.35	12.50 ± 0.07	2.44
Doxorubicin	1.16 ± 0.06	>10	>8.62	Vorinostat	1.14 ± 0.27	2.87 ± 0.57	2.43
Adrucil	15.76 ± 1.52	>50	>3.17	Everolimus	31.52 ± 0.38	>50	>1.58
Abitrexate	30.02 ± 1.68nM	95.92 ± 13.44nM	3.19	Mitotane	58.45 ± 1.97	73.80 ± 0.79	1.26
Bleomycin sulfate	0.26 ± 0.05	>10	>38.46	Azacitidine	46.72 ± 1.04	>100	>2.14
Etoposide	3.38 ± 0.70	44.7 ± 1.26	13.22	Lomustine	77.07 ± 11.83	>100	>1.30
Idarubicin HCl	78.56 ± 8.231nM	>1000nM	>12.72	Tolfenamic acid	53.08 ± 8.195	>100	>1.88
Fludarabine Phosphate	0.21 ± 0.02	>10	>47.61	Mometasone furoate	38.1 ± 4.31	>100	>2.62
2-Methoxyestradiol	1.02 ± 0.01	>10	>9.80	Maraviroc	41.74 ± 3.08	>100	>2.39
Vincristine	<0.019	0.06 ± 0.015	>3.16	Flunarizine 2HCl	7.26 ± 0.09	10.60 ± 1.86	1.46
MDV3100	11.85 ± 0.48	>50	>4.21	Epalrestat	76.40 ± 0.86	>100	>1.31
Floxuridine	3.44 ± 1.047	>50	>14.53	Aspartame	39.74 ± 2.02	>100	>2.53
Mercaptopurine	14.56 ± 2.744	>50	>3.43	Dyclonine HCl	39.75 ± 2.075	>100	>2.51
Ruxolitinib	13.41 ± 3.09	>50	>3.72	Pioglitazone hydrochloride	13.02 ± 1.38	21.44 ± 1.49	1.65
Pralatrexate	0.18 ± 0.09	1.71 ± 1.31	9.50	Bazedoxifene HCl	15.58 ± 1.81	25.04 ± 0.01	1.61
Teniposide	0.14 ± 0.02	18.59 ± 1.99	132.78	OSI-420	1.30 ± 0.007	1.84 ± 0.042	1.41
Diethylstilbestrol	8.12 ± 1.06	27.18 ± 1.06	3.34	Levosimendan	95.47 ± 2.02	>100	>1.04
Fluticasone propionate	0.27 ± 0.02	0.89 ± 0.09	3.30	Clomifene citrate	34.54 ± 0.18	34.38 ± 0.975	1.00
Methacycline hydrochloride	20.44 ± 2.15	>100	>4.89	Mifepristone	30.63 ± 4.57	42.38 ± 0.64	1.38
Phenformin hydrochloride	29.15 ± 1.08	>100	>4.89	Nilvadipine	65.23 ± 1.825	81.57 ± 2.12	1.25
Idebenone	19.11 ± 0.27	>100	>5.23	Rimonabant	15.94 ± 0.08	15.97 ± 0.36	1.00
triamterene	7.31 ± 0.32	72.06 ± 5.24	9.85	Tolcapone	44.85 ± 1.28	>100	>2.23
Irinotecan	42.47 ± 7.38	>50	>1.18	Sertraline HCl	7.9 ± 0.27	10.64 ± 0.25	1.35
Cladribine	7.31 ± 0.94	>10	>1.36	Halobetasol Propionate	52.47 ± 0.44	72.96 ± 1.62	1.39
Clofarabine	19.36 ± 0.16	>50	>2.58	Azaguanine	41.43 ± 2.64	>100	>2.41
Vemurafenib	23.43 ± 0.04	>50	>2.13	Cepharanthine	17.89 ± 2.83	25.04 ± 0.73	1.4
Camptothecin	4.08 ± 0.5	>10	>2.45	Famprofazone	68.55 ± 2.18	>100	>1.46
Fludarabine	0.68 ± 0.06	1.05 ± 0.05	1.54	FK-506	74.72 ± 8.11	>100	>1.33

### Alteration of Cancer Drug Resistance Gene Expression Profile Associated With *BAX* and *BAK* Deficiency in DLD-1 Colon Cancer Cells

**Figure 2:**
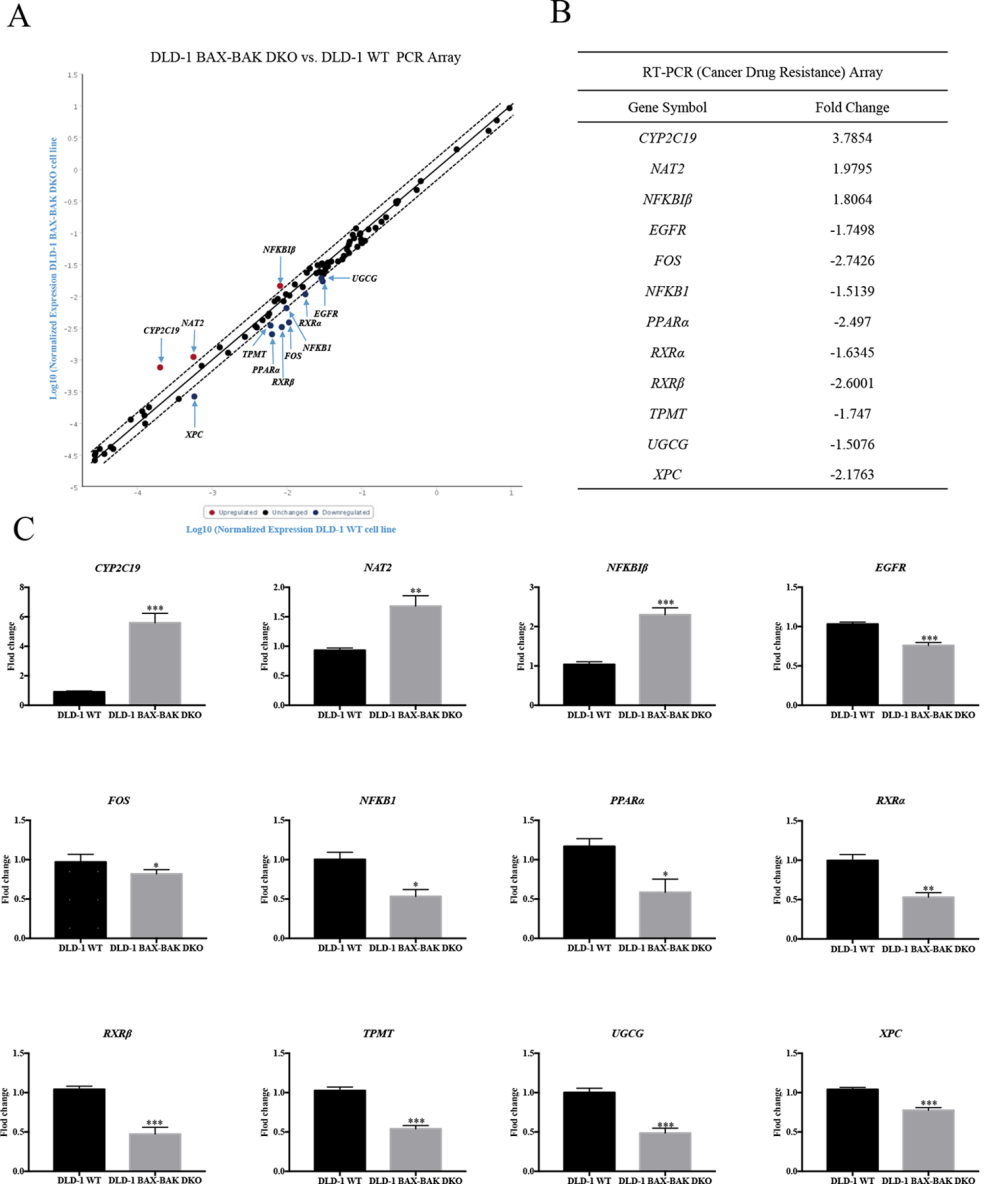
RT² Profiler™ PCR Array analysis for gene expression of DLD-1 BAX–BAK DKO cells. **(A)** Scatter plot of drug resistance genes fold regulation values from DLD-1 BAX–BAK DKO cells relative to DLD-1 WT cells (control). Genes not regulated (black), upregulated genes (red), and downregulated genes (blue) with threshold lines of 1.5 and −1.5. **(B)** List of genes with expression fold change in DLD-1 BAX–BAK DKO cells with more than 1.5-fold up- or down-regulation compared with DLD-1 WT cells. Positive and negative fold changes stand for up- or down-regulation, respectively. **(C)** RT-qPCR independent validation from DLD-1 WT cells (control), or DLD-1 BAX–BAK DKO cells. Gene expressions were normalized to *ACTIN*, relative to control, and analyzed using the 2^−ΔΔCT^ method. The data is represented as mean ± S.D. (n = 3). ***p < 0.001, **p < 0.01 and *p < 0.05, genes expression level in DLD-1 BAX–BAK DKO cells were compared with its level in DLD-1 WT cells, *t*-test analysis.

**Figure 3:**
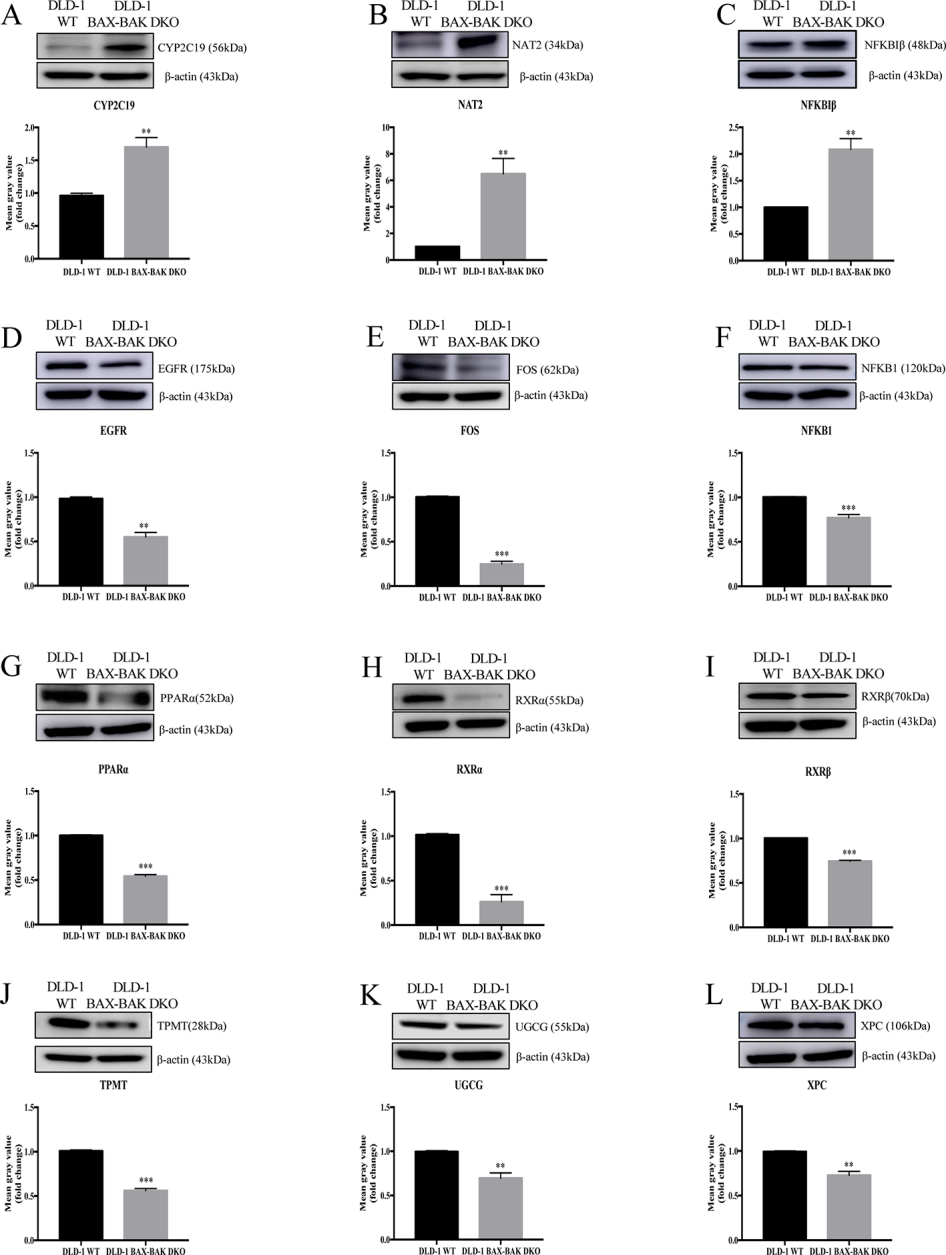
Western blot analysis of relevant proteins expression level in DLD-1 WT and DLD-1 BAX–BAK DKO cells. (**A**–**L**) Cropped images showed protein expression levels of CYP2C19, NAT2, NFKBIβ, EGFR, FOS, NFKB1, PPARα, RXRα, RXRβ, TPMT, UGCG, and XPC in DLD-1 WT and DLD-1 BAX–BAK DKO cells. Bar charts presented relative changes of mean gray value, which was normalized to that of β-actin. The full-length images of Western blot are shown in **Figure S2**. The data is represented as mean ± S.D. (n = 3). ***p < 0.001 and **p < 0.01, proteins expression level in DLD-1 BAX–BAK DKO cells were compared with its level in DLD-1 WT cells, *t*-test analysis.

The effect of *BAX* and *BAK* deletion on the expression levels of other genes related to cancer drug resistance were analyzed by Human Cancer Drug Resistance RT^2^ Profiler™ PCR array assay with DLD-1 cellular models. As shown in the scatter plot (**Figures 2A, B**), 12 out of totally 84 examined genes demonstrated more than 1.5 fold up or down-regulation in the DLD-1 BAX–BAK DKO cells when compared with the wild type counterpart. Amongst which, cytochrome P450 family 2 subfamily C polypeptide 19 (*CYP2C19*), N-acetyltransferase 2 (*NAT2*), and nuclear factor of kappa light polypeptide gene enhancer in B-cells inhibitor beta (*NFKBIβ*) genes were highly upregulated in DLD-1 BAX–BAK DKO cells compared with DLD-1 WT cells. Whereas epidermal growth factor receptor (*EGFR*), FBJ murine osteosarcoma viral oncogene homolog (*FOS*), nuclear factor of kappa light polypeptide gene enhancer in B-cells 1 (*NFKB1*), peroxisome proliferator-activated receptor alpha (*PPARα*), retinoid X receptor alpha (*RXRα*), retinoid X receptor beta (*RXRβ*), thiopurine S-methyltransferase (*TPMT*), UDP-glucose ceramide glucosyltransferase (*UGCG*), and xeroderma pigmentosum, complementation group C (*XPC*) genes were significantly downregulated in DLD-1 BAX–BAK DKO cells. The expression level of these 12 genes were further validated individually by RT-qPCR using primers with sequence as listed in **Table 2**. As shown in **Figure 2C**, the expression profiles of the 12 genes were consistent with the result as presented in the PCR array assay. In addition, the expression levels of the corresponding proteins encoded by these genes as analyzed by Western blot detection demonstrated similar tendency as in the result of gene expression assays (**Figures 3A–L**). Therefore, *BAX* and *BAK* deficiency altered the expression of *CYP2C19*, *NAT2*, *NFKBIβ*, *EGFR*, *FOS*, *NFKB1*, *PPARα*, *RXRα*, *RXRβ*, *TPMT*, *UGCG*, and *XPC* both at the transcriptional and translational levels in DLD-1 BAX–BAK DKO cells. These results were in line with the result in **Table 1** demonstrating that *BAX* and *BAK* are extensively involved in resistance of our cellular model to against a considerable number of FDA-approved drugs with anti-cancer effect.

**Table 2:** RT-qPCR primer sequence.

Gene	Forward primer sequence	Reverse primer sequence
BAX	5’-AAGGTGCCGGAACTGATC-3’	5’-CCGGAGGAAGTCCAATGT-3’
BAK	5’-CCTCCTGCTCCTACAGCA-3’	5’-TGATGCCACTCTCAAACAGG-3’
CYP2C19	5’-GCTGCATGGATATGAAGTGGT-3’	5’-GCTGCATGGATATGAAGTGGT-3’
NAT2	5’-TGGTGTCTCCAGGTCAATCA-3’	5’-CCATGCCAGTGCTGTATTTG-3’
NKFBIβ	5’-GTCTTCGGCTACGTCACTGAG-3’	5’-TGTCTGGCCTAGGTCATTCTG-3’
EGFR	5’-GGTGCAGGAGAGGAGAACTG-3’	5’-GGTGGCACCAAAGCTGTATT-3’
FOS	5’-AGAATCCGAAGGGAAAGGAA-3’	5’-CTTCTCCTTCAGCAGGTTGG-3’
NFKB1	5’-GCCTCTAGATATGGCCACCA-3’	5’-TCAGCCAGCTGTTTCATGTC-3’
PPARα	5’-ACGATTCGACTCAAGCTGGT-3’	5’-GTTGTGTGACATCCCGACAG-3’
RXRα	5’-CAAGGACTGCCTGATTGACA-3’	5’-CTGGTCGACTCCACCTCATT-3’
RXRβ	5’-TCCCACACTTTTCCTCCTTG-3’	5’-AGGATGCCATCTCGAACATC-3’
TPMT	5’-ACGGCAAGACTGCTTTTCAT-3’	5’-CTACACTGTGTCCCCGGTCT-3’
UGCG	5’-CAATGCAAAACTCTGGCTCA-3’	5’-GAACCAGGCGACTGCATAAT-3’
XPC	5’-CCATGAGGACACACACAAGG-3’	5’-TCCAATGAACCACTTCACCA-3’

### Knockdown of *XPC* Suppresses the Sensitivity to Etoposide in DLD-1 Colon Cancer Cells

**Figure 4:**
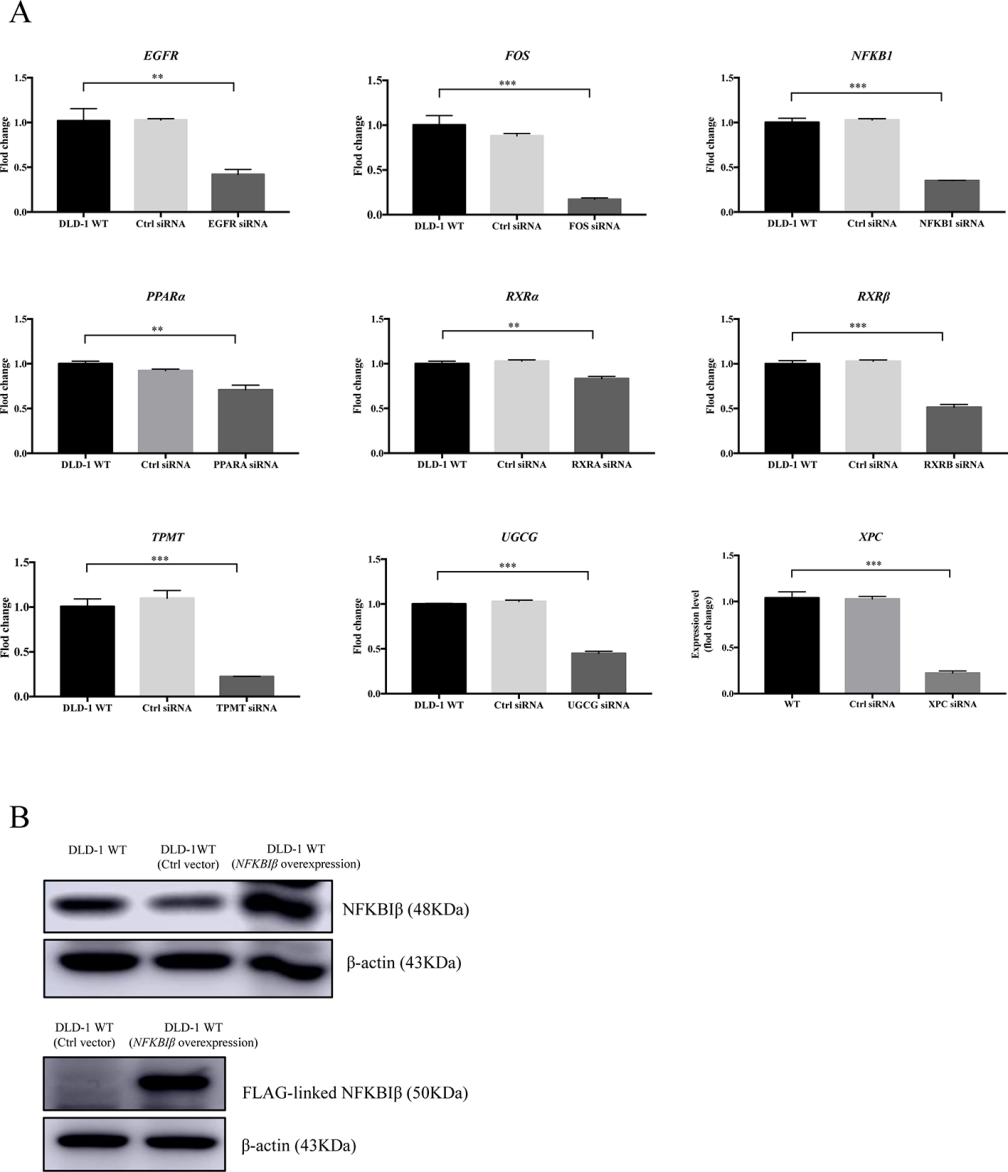
Expression level of specific gene or protein in DLD-1 WT cells after siRNA-mediated gene silencing or protein overexpression. **(A)** The expression levels of the targeted genes knockdown by specific siRNA in DLD-1 WT cell, DLD-1 ctrl siRNA cells and DLD-1 specific gene knockdown cells. **(B)** NFKBIβ and FLAG-linked NFKBIβ proteins expression levels in DLD-1 WT, DLD-1 ctrl vector cells, and DLD-1 WT cells transfected with FLAG-HA-tagged *NFKBIβ* plasmid. β-actin was used as internal control. The full-length images of Western blot are shown in **Figure S3**. The data is represented as mean ± S.D. (n = 3). ***p < 0.001, **p < 0.01 were compared as indicated, *t*-test analysis.

Based on the gene array and Western blot data, the 9 downregulated genes (*EGFR*, *FOS*, *NFKB1*, *PPARα*, *RXRα*, *RXRβ*, *TPMT*, *UGCG*, and *XPC*) in the DLD-1 BAX–BAK DKO cells potentially involved in BAX/BAK-mediated drug resistance were further investigated by downregulating their endogenous expression in the DLD-1 WT cells with siRNA-mediated gene silencing. Control siRNA were used as blank controls and the transfection efficiency was verified by RT-qPCR detection. As shown in **Figure 4A**, the gene expression levels of all the siRNA-transfected cells were significantly downregulated when compared with DLD-1 WT cells and DLD-1 ctrl siRNA cells. Amongst the 3 upregulated genes, *CYP2C19* and *NAT2* have long been reported to participate in the failure of anticancer agents treatment or the increased occurrence of cancers (Hein et al., 1993; Hein et al., 2000; Rochat, 2005; AbuHammad and Zihlif, 2013). In contrast, literature concerning *NFKBIβ* and the development of cancer drug resistance or tumorigenesis is scarce, therefore, the gene was selected for clarifying its mechanistic role in the development of drug resistance. *NFKBIβ* was examined by enhancing the expression of the gene *via* transfection of FLAG-HA-tagged *NFKBIβ* plasmid (FLAG_NFKBIβ_) into DLD-1 WT cells. The expression level of basal NFKBIβ and FLAG-linked NFKBIβ proteins in the DLD-1 WT cells transfected with FLAG_NFKBIβ_ (DLD-1 NFKBIβ overexpression cells) or FLAG-HA tag only (DLD-1 ctrl vector cells) were recognized by NFKBIβ and FLAG antibodies, respectively. As illustrated in **Figure 4B**, the protein expression of FLAG-linked NFKBIβ significantly increased after FLAG_NFKBIβ_ transfection when compared with the basal NFKBIβ level as observed in DLD-1 ctrl vector cells and DLD-1 WT cells, suggesting that the transfection was successful. To further confirm whether the altered expression of these 10 genes (*NFKBIβ*, *EGFR*, *FOS*, *NFKB1*, *PPARα*, *RXRα*, *RXRβ*, *TPMT*, *UGCG*, and *XPC*) in DLD-1 colon cancer cells would affect their responsiveness towards chemotherapeutics agents, MTT assay was performed to analyze the viability of the transfected cells after etoposide treatment. As presented in **Table 3**, DLD-1 WT cells knockdown with siRNA for *XPC* (DLD-1 siXPC cells) showed the highest IC_50_ value (> 60 μmol) and resistance (RF = 18.23) towards etoposide compared with the DLD-1 WT cells. The response of *XPC* knockdown was comparable to those observed in DLD-1 BAX–BAK DKO cells (IC_50_ value > 40 μmol; RF = 13.22) suggesting the enhancing role of *XPC* in the development of resistance in DLD-1 colon cancer cells model against chemotherapy.

**Table 3:** Cytotoxicity of etoposide was detected in DLD-1 WT, DLD-1 BAX–BAK DKO, and DLD-1 WT cells transfected with siRNA or with *NFKBIβ* overexpression.

Cells line	IC_50_ value (µmol)	Resistance fold
DLD-1 WT cells	3.38 ± 0.70	–
DLD-1 BAX–BAK DKO cells	44.7 ± 1.26	13.22
DLD-1 siXPC cells	61.64 ± 5.45	18.23
DLD-1 NFKBIβ overexpression cells	33.76 ± 0.92	9.98
DLD-1 siEGFR cells	9.74 ± 2.88	2.88
DLD-1 siRXRβ cells	9.71 ± 2.87	2.87
DLD-1 siRXRα cells	7.37 ± 1.70	2.18
DLD-1 siFOS cells	5.58 ± 1.65	1.65
DLD-1 siTPMT cells	4.06 ± 1.20	1.20
DLD-1 siPPARα cells	2.88 ± 0.85	0.85
DLD-1 siUGCG cells	2.33 ± 0.69	0.69
DLD-1 siNFKB1 cells	1.52 ± 0.45	0.45

### *BAX* and *BAK* Deficiency Upregulated Mitochondrial Respiration and Glycolysis in DLD-1 Colon Cancer Cells

**Figure 5:**
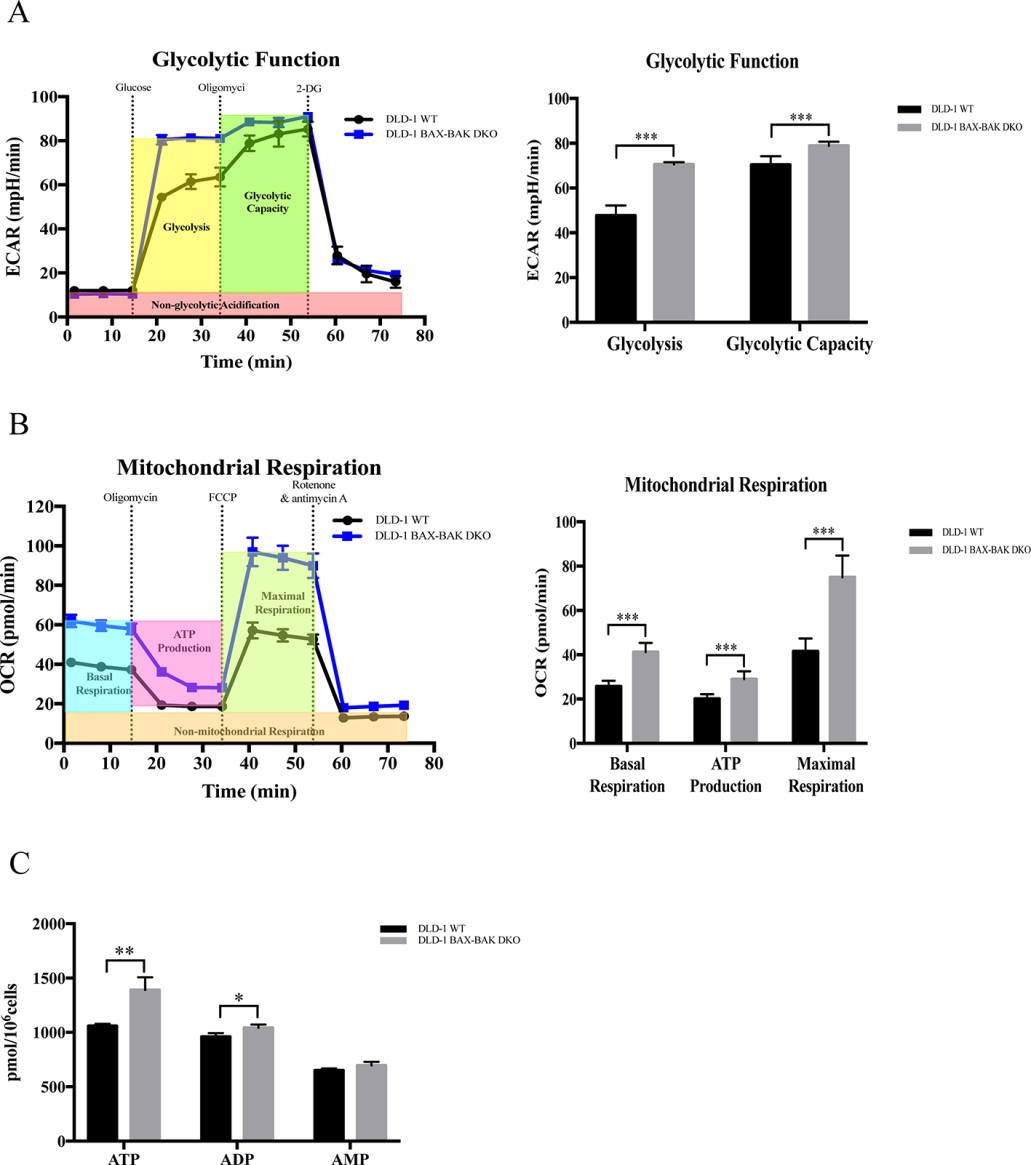
Capacity of glycolytic and mitochondrial respiration in DLD-1 WT and DLD-1 BAX–BAK DKO cells. **(A)** The glycolytic profile of DLD-1 WT and DLD-1 BAX–BAK DKO cells. The glycolytic function was measured by directly detecting the ECAR of cells. The compounds (glucose, oligomycin, 2-DG) were serially injected to measure the glycolysis and glycolytic capacity, respectively. **(B)** The mitochondrial respiration profile of DLD-1 WT and DLD-1 BAX–BAK DKO cells. Mitochondrial respiration test determines the key parameters of mitochondrial function by directly measuring the OCR of cells. The compounds (oligomycin, FCCP, and a mix of rotenone and antimycin A) were serially injected to measure the basal respiration, ATP production, and maximal respiration, respectively. **(C)** ATP production (pmol/10^6^ cells) in DLD-1 WT and DLD-1 BAX–BAK DKO colon cancer cells. The data is represented as mean ± S.D. (n = 3). ***p < 0.001, **p < 0.01 and *p < 0.05 compared with DLD-1 WT cells, *t*-tests analysis.

Upregulation of glycolysis in cancer cells would facilitate their survival during energy deprivation resulting in the acquisition of therapeutic resistance (Correia and Bissell, 2012; Bhattacharya et al., 2016). Therefore, we clarified the cellular energy metabolic activity of both DLD-1 WT and DLD-1 BAX–BAK DKO cells. The metabolisms of these cancer cells were characterized by the use of Seahorse XFp analyzer by comparing with the DLD-1 WT cells. Firstly, DLD-1 BAX–BAK DKO and DLD-1 WT cells were glucose-starved for 1h before the start of experiments. The glycolytic activity of the cellular model was assessed based on their ECAR. Since glycolytic metabolism is a dynamic process, glycolysis will be inhibited through glucose deprivation and set as zero before the experiment. As presented in **Figure 5A**, the basal glycolysis (after the administration of saturated glucose solution), and glycolytic capacity (after the suppression of the ATP synthase by oligomycin) were significantly increased in DLD-1 BAX–BAK DKO cells when compared with DLD-1 WT cells. 2-DG was injected at the last step to inhibits glycolysis *via* competitive combining to glucose hexokinase. Finally, under the inhibition of 2-DG, ECAR value decreased to the same level as the experiment started, substantiating that the ECAR induction during the reaction was entirely caused by glycolysis. Such result indicated the prominent role of BAX and BAK in the regulation of glycolysis in DLD-1 cellular models because the basal glycolysis and glycolytic capacity were significantly increased in DLD-1 BAX–BAK DKO cells when compared with DLD-1 WT cells. Subsequently, the effect of *BAX* and *BAK* double knockout in the mitochondrial respiration rates in DLD-1 colon cancer cells was analyzed by measuring the OCR of DLD-1 cellular models with the use of different inhibitors or stimulators of the mitochondrial respiratory pathway. As demonstrated in **Figure 5B**, the cellular basal respiration, ATP production (after the inhibition of the ATP synthase by oligomycin), maximal respiration (after the stimulation of the OCR by FCCP), and non-mitochondrial respiration (after the mitochondrial respiration shuts down by the combination of rotenone and antimycin A) were significantly increased in DLD-1 BAX–BAK DKO cells when compared with DLD-1 WT cells. In addition, LC-MS/MS-based ATP metabolites measurement showed that DLD-1 BAX–BAK DKO cells generated more ATP than DLD-1WT cells (**Figure 5C**). Therefore, deficiency of *BAX* and *BAK* elevated cellular energy metabolism *via* the upregulation of mitochondrial oxidative respiratory reaction and glycolysis, suggesting that the glucose catabolism was involved in the development of drug resistance.

### Silencing of *XPC* Promoted Glycolysis in DLD-1 Colon Cancer Cells

**Figure 6:**
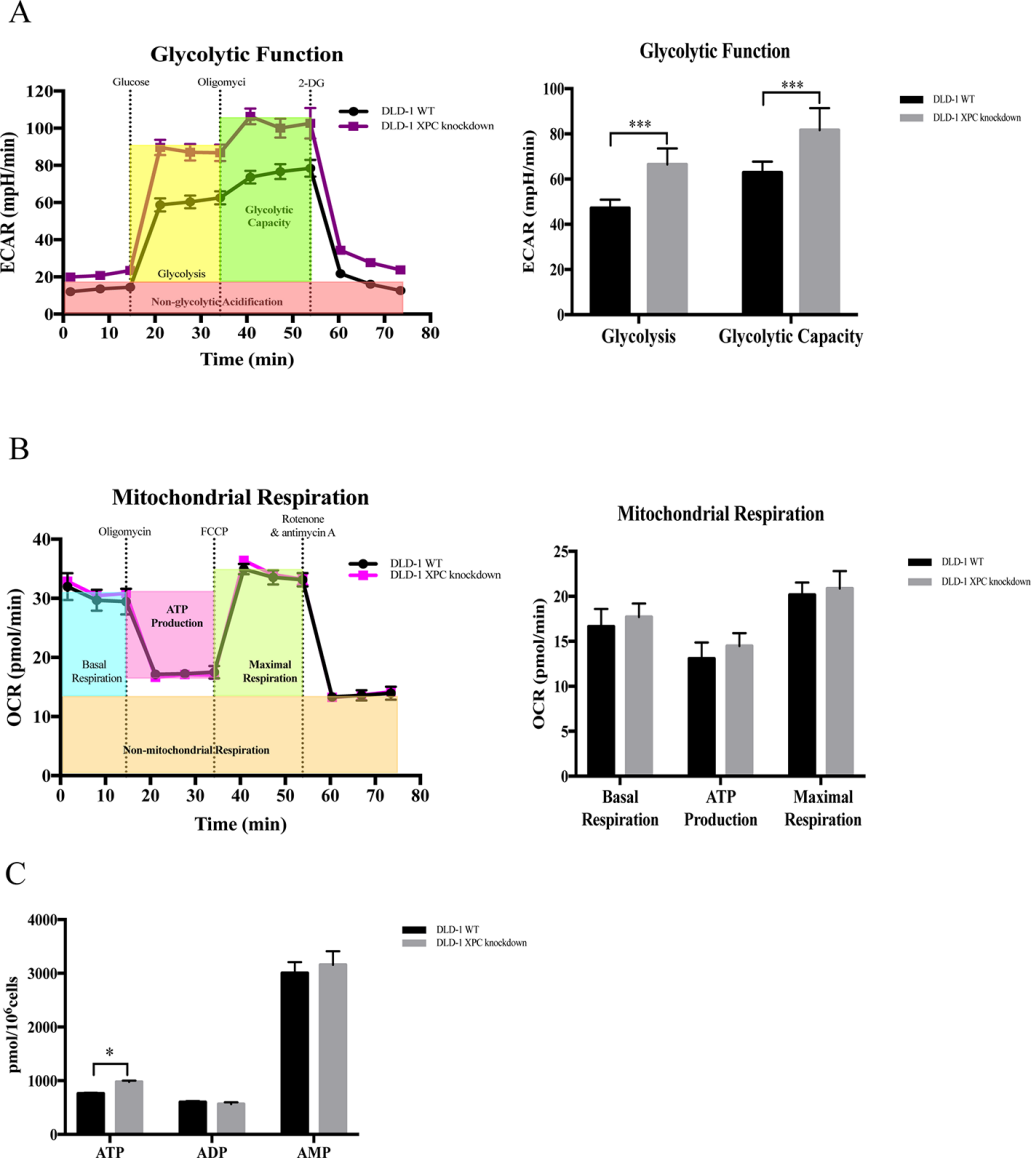
Capacity of glycolytic and mitochondrial respiration in DLD-1 WT cells and DLD-1 siXPC cells. **(A)** The glycolytic profile of DLD-1 WT and DLD-1 siXPC cells. **(B)** The mitochondrial respiration profile of DLD-1 WT and DLD-1 siXPC cells. **(C)** ATP production (pmol/10^6^ cells) in DLD-1 WT and DLD-1 siXPC cells. The data is represented as mean ± S.D. (n = 3). ***p < 0.001 and *p < 0.05 compared with DLD-1 WT cells, *t*-tests analysis.

Evidence showed that silencing of *XPC* would promote glycolysis (Rezvani et al., 2011a). In current research, the expression level of *XPC* gene was downregulated in DLD-1 colon cancer cells with the deficient of both *BAX* and *BAK*. Therefore, to verify if the upregulation of cellular energy metabolism and the drug resistance observed in DLD-1 BAX–BAK DKO cells was associated with the suppression of *XPC* gene, seahorse analysis was used to assess the ECAR and OCR in DLD-1 siXPC cells and DLD-1 WT cells. As presented in **Figure 6A**, the basal glycolytic and maximal glycolytic capacity of DLD-1 *XPC* knockdown cells were significantly higher than that of DLD-1 WT cells. These data indicated that *XPC* gene silencing would stimulate the glycolysis in DLD-1 colon cancer cells. However, as shown in **Figure 6B**, the mitochondrial respiratory response of DLD-1 siXPC cells demonstrated no significant alteration after stimulation with oligomycin and FCCP as compared with DLD-1 WT cells. In addition, when compared with DLD-1 WT cells, the accumulation of ATP in DLD-1 siXPC cells were significantly enhanced to a small extent by the downregulation of *XPC* (**Figure 6C**). In conclusion, the downregulation of *XPC* plays an important role in drug resistance acquirement *via XPC*-mediated upregulation of glycolysis.

## Discussion

Chemotherapy represents one of the mainstays of pharmacological intervention for cancer treatment. The mechanism of action (MOA) for most anticancer drugs, for example, platinum-based compounds and topoisomerase inhibitors, involves the induction of DNA damage, the downstream apoptosis-mediated cytotoxicity, and microenvironment variation (Longley and Johnston, 2005; Curtin, 2012). Therefore, repaired DNA damage, hampered apoptotic machinery of cancer cells, together with altered microenvironment during the course of tumorigenesis, are critical to the development of drug-resistant phenotype, which remains a major concern for therapeutic efficacy (Taylor et al., 2000; Longley and Johnston, 2005). In particular, CRC is interested in this study due to the low survival rate of patients resulted from poor diagnosis (Vega et al., 2015). The invasive colonoscopy for the standard assessment of CRC discouraged patients from early diagnosis. In addition, there are no specific symptoms for CRC and common abdominal symptoms are mostly unrelated to the occurrence of neoplasm, patients are usually diagnosed at the advanced stage of the disorder demonstrating significant chemoresistance (Van Der Jeught et al., 2018).

In this study, we investigated the mechanism underpinning the chemoresistance of DLD-1 BAX–BAK DKO colon cancer cells. Research finding suggested that *Bax*-deficiency alone promotes etoposide resistance in MEFs (McCurrach et al., 1997), while the overexpression of *BAK* confers sensitivity of drug-resistant upon MT1-Adr breast carcinoma (von Haefen et al., 2002). However, Wei et al. reported that MEFs demonstrate resistance to etoposide and ultraviolet radiation only when these cells are knocked out with both *Bax* and *Bak* (Wei et al., 2001). As such, DLD-1 BAX–BAK DKO cells were used in this study for establishing a cellular model with reliable resistant phenotype against chemotherapeutic drug-induced apoptosis. Our findings showed that DLD-1 BAX–BAK DKO cells were relatively insensitive to etoposide-induced apoptosis when compared to wild type control, confirming the drug-resistant nature of our cellular model.

After the screening of more than 200 FDA-approved drugs against the DLD-1 cells models, 66 of them were found capable of inducing cytotoxicity towards the DLD-1 WT cells (i.e. IC_50_ < 100 μmol). The DLD-1 BAX–BAK DKO cells are resistant (RF ≥ 3) to 41% (27/66) and sensitive (RF < 3) to the rest (59%; 39/66) of the 66 drugs relative to the wide type counterpart. The induced cytotoxicity in the apoptosis-resistant DLD-1 BAX–BAK DKO cells is most probably due to the activation of cells death pathways other than apoptosis. For example, sorafenib which demonstrated the RF value of 1.08 in this study are having the capacity to trigger autophagic cells death in hepatocellular carcinoma and gastrointestinal cancer cells mediated by the inhibition of myeloid cells leukemia-1 (MCL-1) and activation of CD95, respectively (Park et al., 2010; Tai et al., 2013). In fact, our previous findings suggested that cytotoxicity can be induced in DLD-1 BAX–BAK DKO cells *via* the activation of autophagic cells death *via* the manipulation of 5’ AMP-activated protein kinase (AMPK) (Law et al., 2016; Law et al., 2017). However, some of the tested drugs with RF ≥ 3 are also inducer of autophagic cells death, such as, dasatinib (RF > 23.25) through the mediation of beclin1 and Akt in ovarian cancer (Le et al., 2010). Such diverse behavior of DLD-1 BAX–BAK DKO cells could be explained by the involvement of different molecular pathways mediating the induction of autophagy by these drugs, and that the activation of autophagy is highly cells type-specific. In-depth analysis of the MOA of the 27 FDA-approved drugs demonstrating insensitivity towards DLD-1 BAX–BAK DKO cells provided insight to illustrate the molecular details underlying the observed chemoresistance. Twenty-three out of these 27 drugs are conventional anticancer pharmaceuticals acting against cancers through the cellular processes, including the inhibition of angiogenesis, antiproliferation, and the modulation of extrinsic and intrinsic pathways of apoptosis. The remaining 4 drugs, including fluticasone propionate, phenformin hydrochloride, idebenone, and triamterene, are suggested treatment for metabolic disorders or inflammation by the FDA. Accordingly, the DLD-1 BAX–BAK DKO cells may circumvent the cytotoxicity induced by these 27 drugs, at least partially, owing to their apoptosis-deficient nature. Indeed, the involvement of other cellular responses in DLD-1 BAX–BAK DKO cells associated with the resistance to these drugs is far more complex and cannot be neglected.

Therefore, it was tempted to clarify the downstream effects of *BAX* and *BAK* deficiency in the development of cancer drug resistance. From our gene array study, *BAX* and *BAK* deficiency in DLD-1 BAX–BAK DKO cells significantly downregulated the expression of *XPC* compared with DLD-1 WT cells. Of note, our findings suggest that DLD-1 WT cells transfected with siRNA of XPC illustrated the highest resistance upon cytotoxicity assay. In principle, downregulation of *XPC* can enhance apoptosis by hampering the efficiency of NER. However, our finding was contradictory to the previous studies, since the damaged NER may be compensated through other DNA repair pathways in DLD-1 siXPC cells. We then investigated the functional effects of XPC which may potentially lead to the development of chemoresistance in the DLD-1 BAX–BAK DKO cells. Apart from participation to DNA repairment, XPC is critical to the maintenance of cellular energy metabolism (Rezvani et al., 2011a). For example, transduction of siRNA of XPC into human keratinocytes induces lactic acidosis which resulted in suppressed expression of mitochondrial OXPHOS subunits and upregulation of enzymes associated with glycolysis (Rezvani et al., 2011b). Our data suggested that both knockout of *BAX*/*BAK* or knockdown of *XPC* affected glycolysis in DLD-1 WT cells. The altered expression of XPC may affect the sensitivity of DLD-1 colon cancer cells to etoposide by manipulating the glycolytic process, since cancer cells have the preference for acquiring cellular energy from aerobic glycolysis over the usual oxidative phosphorylation pathway. In addition, the increased level of glycolysis metabolites can facilitate the biosynthesis of nucleotides, lipid, and macromolecules which promotes the unconstrained proliferation of cancer cells (Gatenby and Gillies, 2004; Heiden et al., 2009; Phan et al., 2014). Intriguingly, the expression of the proto-oncogenes responsible for cell growth and proliferation, including *KRAS*, *HRAS*, *NRAS*, *MYB*, and platelet derived growth factor subunit B (PDGFB), in DLD-1 BAX–BAK DKO cells were suppressed when compared with the WT control (**Figure S4**), which suggested that the development of drug-resistant phenotype may not necessary be associated with the proliferation ability of the cancer cells, implicating the critical role of apoptosis resistance in such issue.

Taken together, *BAX* and *BAK* deficiency in the DLD-1 colon cancer cells downregulated the expression of *XPC* and altered the energy metabolism of cancer cells *via* the upregulation of glycolysis implicating the development of drug resistance. Since the use of combination of drugs is usual therapeutic strategy for the treatment of colon cancers, our findings suggested that compounds which are capable of modulating the glycolytic pathway are potential pharmacologic candidates for simultaneous use with other conventional drugs, in particularly, for colon cancers resistant to chemotherapy. As such, treatment assay with the use of xenograft models deserves further in-depth investigation. The current findings identified and confirmed the genes associated with drug-resistant DLD-1 colon cancers may provide insight to the establishment of xenograft model for examining the *in vivo* efficacy of compounds with therapeutic potential. Provided that, BAX and BAK are clinically relevant to the pathogenesis of colorectal cancer (Krajewska et al., 1996; Ogura et al., 1999; Bandrés et al., 2006), the findings of this study encourage to the search of novel prognostic and therapeutic strategies targeting the glycolytic pathway mediated by the BAX/BAK-XPC axis for improving the therapy of colon cancer, particularly for patients demonstrating the resistant phenotype. The activated genes identified by the cancer drug resistance array could serve as reference markers to assess the efficacy of targeted therapy specifically for the treatment of colon cancers with drug-resistant phenotype.

## Data Availability

The raw data supporting the conclusions of this manuscript will be made available by the authors, without undue reservation, to any qualified researcher.

## Author Contributions

VW, BL and LL conceived and designed the research; YH and SM drafted the manuscript; YH, YQ, JC, CX, HH, ZL, CQ, and WZ performed experiments; YH, JC, and YQ analyzed data; SM and YQ edited the article; VW, BL and LL approved the final version of the article.

## Conflict of Interest Statement

The authors declare that the research was conducted in the absence of any commercial or financial relationships that could be construed as a potential conflict of interest.

## Abbreviations

CRC, Colorectal cancer; DLD-1 WT cells, DLD-1 wild-type colon cells; DLD-1 BAX–BAK DKO cells, DLD-1 *BAX* and *BAK* double knockout colon cells; NER, Nucleotide excision repair; MEFs, Mouse embryonic fibroblasts; RT-qPCR, Real-Time quantitative PCR; FDA, Food and Drug Administration; MTT, 3-(4,5-dimethylthiazol-2-yl)-2,5-diphenyltetrazolium bromide; RF, Resistance fold; IC, Cells viability and inhibitory concentration; OCR, Oxygen consumption rate; ECAR, Extracellular acidification rate; FCCP, Carbonyl cyanide 4-(trifluoromethoxy) phenylhydrazone; OXPHOS, Oxidative phosphorylation; 2-DG, 2-deoxyglucose; MOA, Mechanism of action; *CYP2C19*, Cytochrome P450 family 2 subfamily C polypeptide 19; *NAT2*, N-acetyltransferase 2; *NFKBIβ*, Nuclear factor of kappa light polypeptide gene enhancer in B-cells inhibitor beta; *EGFR*, Epidermal growth factor receptor; *FOS*, FBJ murine osteosarcoma viral oncogene. homolog; *NFKB1*, Nuclear factor of kappa light polypeptide gene. enhancer in B-cells 1; *PPARα*, Peroxisome proliferator-activated receptor alpha; *RXRα*, Retinoid X receptor alpha; *RXRβ*, Retinoid X receptor beta; *PDGFB*, Platelet derived growth factor subunit B; *TPMT*, Thiopurine S-methyltransferase; *UGCG*, UDP-glucose ceramide glucosyltransferase; *XPC*, Xeroderma pigmentosum complementation group C; FLAG_NFKBIβ_, FLAG-HA-tagged *NFKBIβ* plasmid; DLD-1 NFKBIβ overexpression cells, DLD-1 Wild-type cells *NFKBIβ* overexpression; DLD-1 ctrl vector cells, DLD-1 Wild-type cells transfected with FLAG- HA tag only; DLD-1 ctrl siRNA cells, DLD-1 Wild-type cells silencing with control siRNA; DLD-1 siEGFR cells, DLD-1 Wild-type cells *EGFR* knockdown; DLD-1 siFOS cells, DLD-1 Wild-type cells *FOS* knockdown; DLD-1 siNFKB1 cells, DLD-1 Wild-type cells *NFKB1* knockdown; DLD-1 siPPARα cells, DLD-1 Wild-type cells *PPARα* knockdown; DLD-1 siRXRα cells, DLD-1 Wild-type cells *RXRα* knockdown; DLD-1 siRXRβ cells, DLD-1 Wild-type cells *RXRβ* knockdown; DLD-1 siTPMT cells, DLD-1 Wild-type cells *TPMT* knockdown; DLD-1 siUGCG cells, DLD-1 Wild-type cells *UGCG* knockdown; DLD-1 siXPC cells, DLD-1 Wild-type cells *XPC* knockdown.
