# Supplementary material for: A Novel Drug Resistance Mechanism: Genetic Loss of Xeroderma Pigmentosum Complementation Group C (XPC) Enhances Glycolysis-Mediated Drug Resistance in DLD-1 Colon Cancer Cells
Source: Front Pharmacol. 2019 Sep 10;10:912. doi: 10.3389/fphar.2019.00912 (PMC6746939; doi:10.3389/fphar.2019.00912)
Supplement: Supplementary file 1 [file DataSheet_1.pdf]

frontiers

A Novel Drug Resistance Mechanism: Genetic Loss of Xeroderma Pigmentosum Complementation Group C (*XPC*) Enhances Glycolysis-Mediated Drug Resistance in DLD-1 Colon Cancer Cells

Yu Han[1], Yuan Qing Qu[1], Simon Wing Fai Mok[1], Juan Chen[2], Cheng Lai Xia[3], Hu Qiang He[1], Zheng Li[1], Wei Zhang[1], Cong Ling Qiu[1], Liang Liu[1*], Betty Yuen Kwan Law[1*] and Vincent Kam Wai Wong[1*]

[1]State Key Laboratory of Quality Research in Chinese Medicine, Macau University of Science and Technology, Avenida Wai Long, Taipa, Macau, China

[2]The Key Laboratory of Molecular Biology of Infectious Diseases designated by the Chinese Ministry of Education, Chongqing Medical University

[3]Foshan Maternal and Child Health Research Institute, Foshan Women and Children's Hospital Affiliated to Southern Medical University, Foshan 528000, China

[*]Corresponding authors: Prof. Liang Liu, Dr. Betty Yuen Kwan Law, and Dr. Vincent Kam Wai Wong

Address correspondence: State Key Laboratory of Quality Research in Chinese Medicine, Macau University of Science and Technology, Avenida Wai Long, Taipa, Macau, China

E-mail address: lliu@must.edu.mo (L Liu), yklaw@must.edu.mo (BYK Law); bowaiwong@gmail.com (VKW Wong)

Supplementary Figures

Figure S1

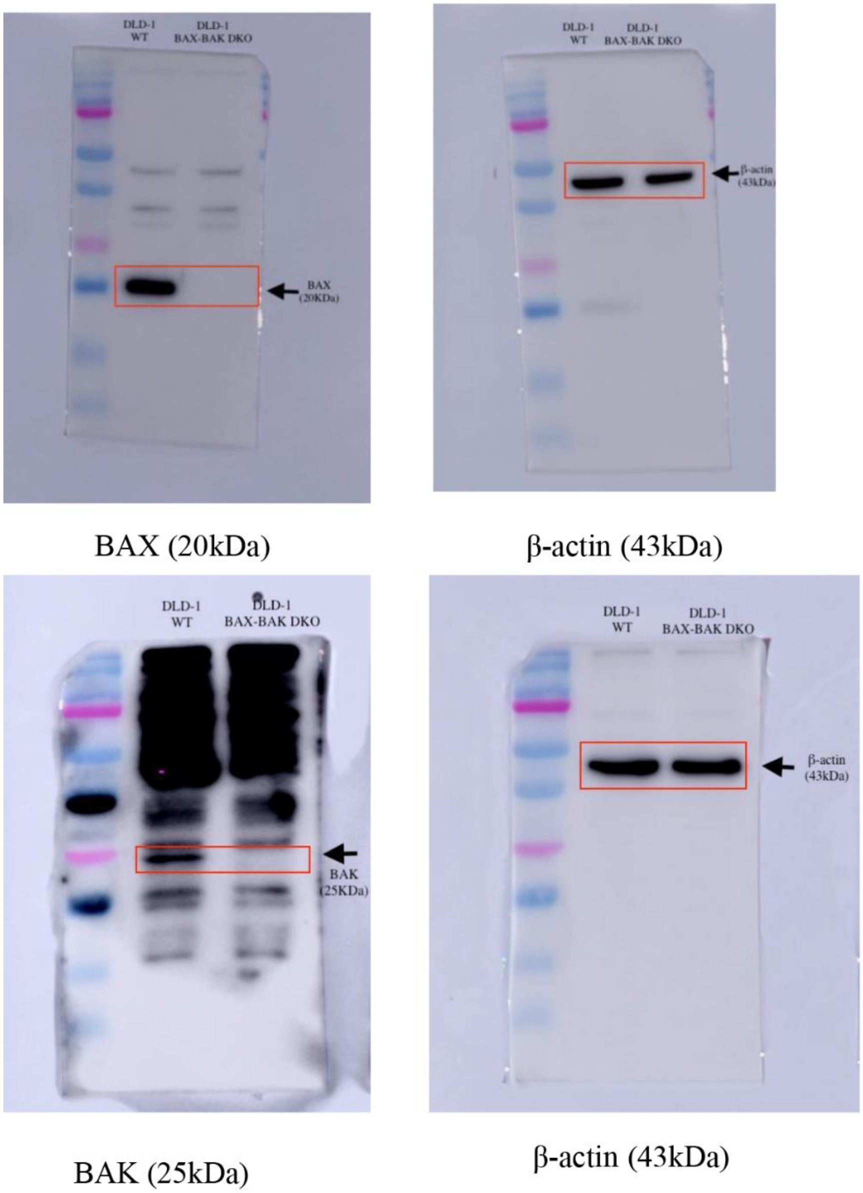

Figure 1 Study of BAX and BAK expression level in DLD-1 colon cancer cells. The full-length images of Western blot are shown in **Figure S1.**

Figure S2

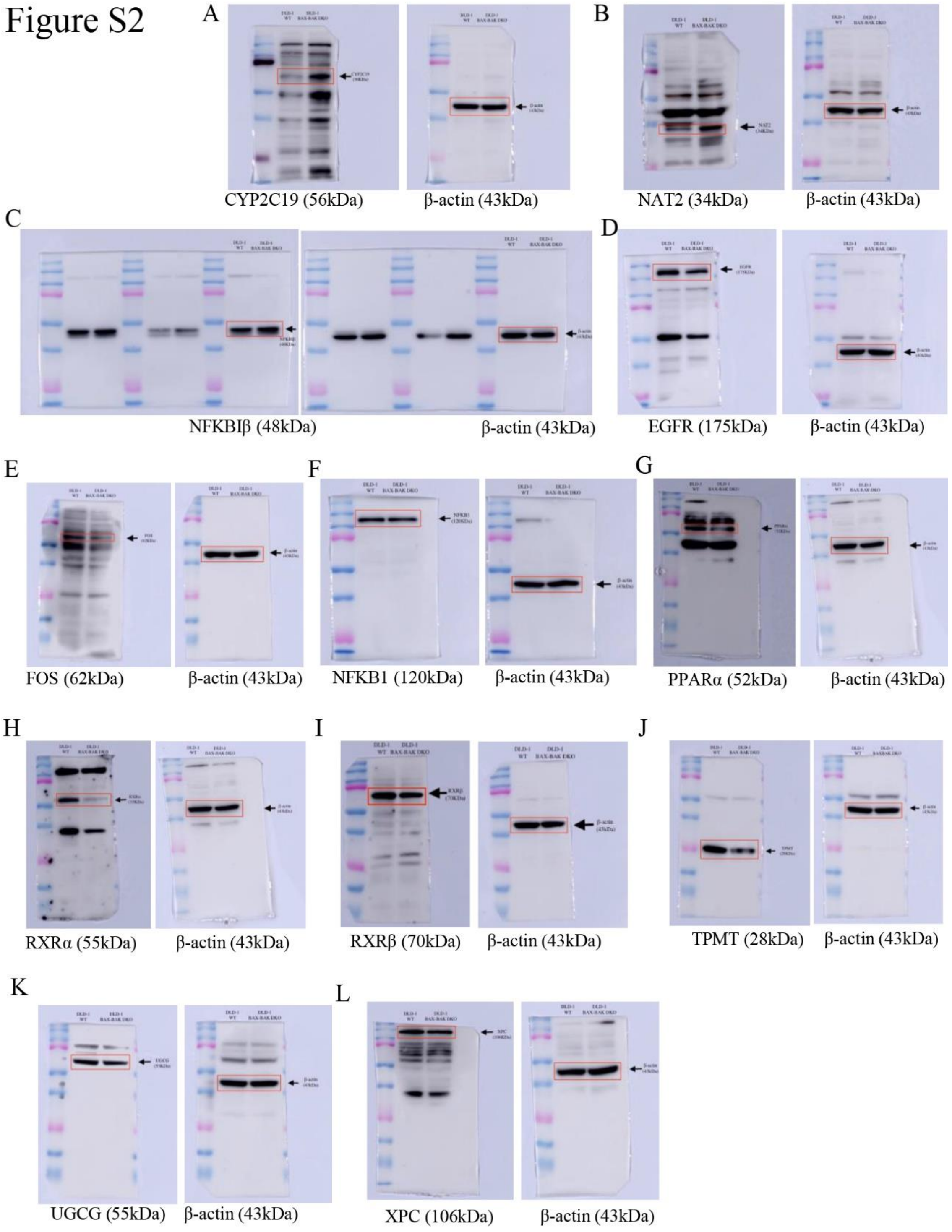

Figure 3 Western blot analysis of relevant proteins expression level in DLD-1 WT and DLD-1 BAX–BAK DKO cells. The full-length images of Western blot are shown in **Figure S2**.

Figure S3

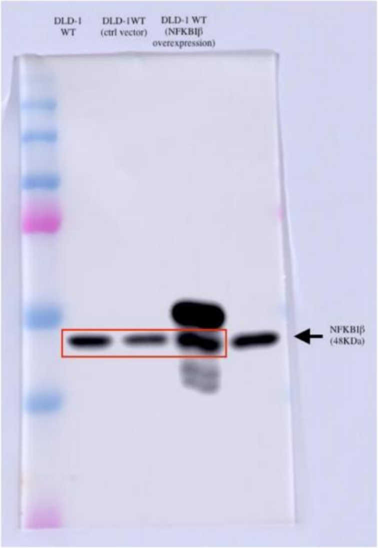

NFKBIβ (48kDa)

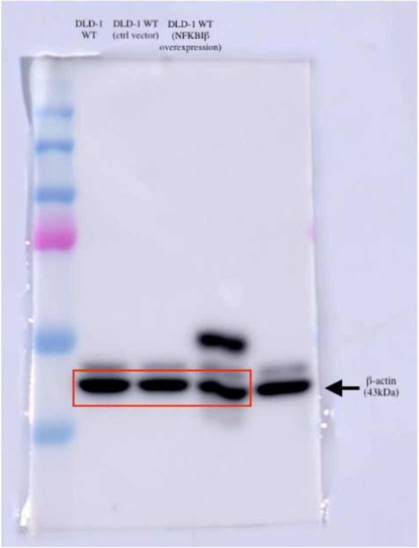

β-actin (43kDa)

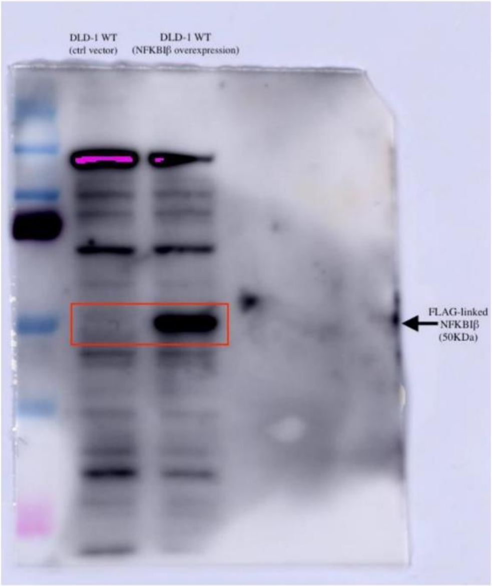

FLAG-linked-NFKBIβ (50kDa)

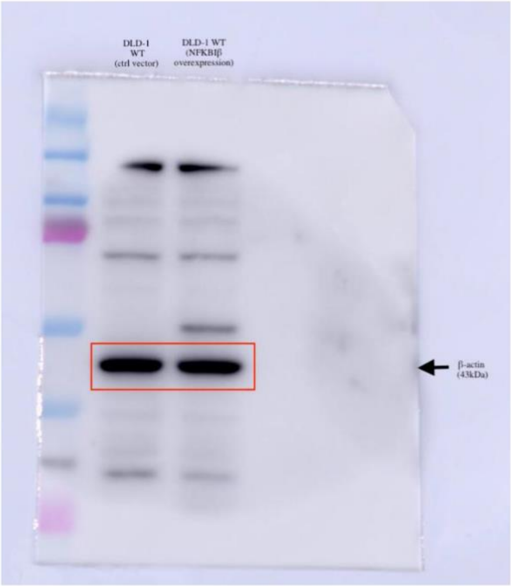

β-actin (43kDa)

Figure 4 Expression level of specific gene or protein in DLD-1 WT cells after siRNA-mediated gene silencing or protein overexpression. The full-length images of Western blot are shown in **Figure S3**.

Figure S4

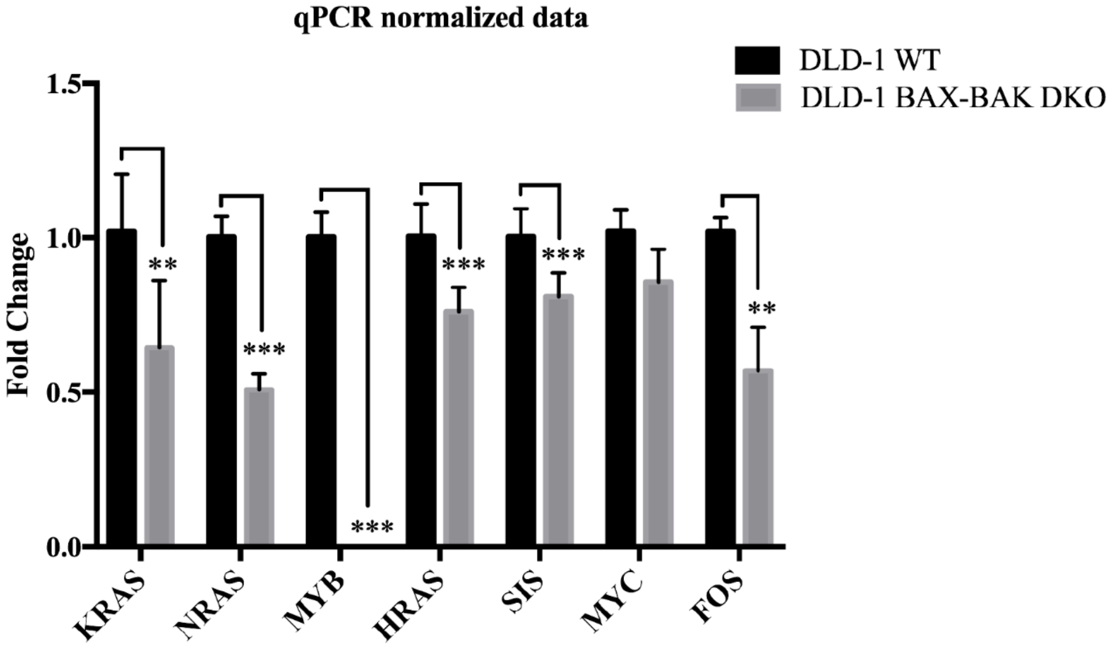

Figure S4. The normalized expression level (compared with DLD-1 WT cells) of inherent mutated oncogenes: MYC, KRAS, NRAS, MYB, HRAS, SIS and FOS in DLD-1 BAX/BAK double-knockout cells.
